# Professionalism on camera: results of a telehealth etiquette pilot curriculum on health profession students’ competency

**DOI:** 10.3389/frhs.2026.1840148

**Published:** 2026-05-25

**Authors:** Rachel Pittmann, Esther Herring, Suzanne Pennington, Rachel Simms, Kathy Sabo

**Affiliations:** 1 Center for Interprofessional Education & Practice, MGH Institute of Health Professions, Boston, MA, United States; 2 Department of Communication Sciences & Disorders, MGH Institute of Health Professions, Boston, MA, United States; 3 School of Nursing, MGH Institute of Health Professions, Boston, MA, United States

**Keywords:** digital readiness, health profession students, kolb experiential learning theory, simulated participant, simulation, telehealth, telehealth etiquette, webside manner

## Abstract

**Introduction:**

Telehealth etiquette refers to professional behaviors that support effective virtual communication between patients and providers. While telehealth has become a lasting part of healthcare delivery, most health professions programs have been slow to respond to the changing healthcare landscape through curricular updates. This pilot study explored the impact of a novel telehealth etiquette curriculum on graduate health professions students' competencies and on patient satisfaction ratings utilizing proxy ratings from simulated participants (SP).

**Methods:**

Using a mixed-methods design, eight graduate students (four nurse practitioner, four speech-language pathology) participated in the curriculum, inclusive of asynchronous training, precepted practice, telesimulations with an SP, and weekly self-reflections in a student-led university clinic. Pre- and post-intervention data included student surveys and weekly journal reflections as well as SP ratings.

**Results:**

Students increased their application of etiquette skills significantly (*z* = 2.52, *p* = .008, *r* = .892), reflecting an 18% improvement based on the Telehealth Etiquette Competency Checklist. Analysis of qualitative content from weekly student self-reflections revealed that eye contact, nonverbal empathy, environmental control, and structured visit closure were the telehealth etiquette skills most positively influencing client–clinician relationships. All students noted that applying telehealth etiquette skills fostered positive relationships with their clients when conducting virtual sessions. SP satisfaction ratings did not significantly change.

**Discussion:**

The telehealth etiquette curriculum improved students' growth in patient-centered videoconferencing-based virtual care communication, thereby optimizing the digital readiness of these future health professionals. Although improvements in proxy patient satisfaction were not found, students consistently described stronger connections with clients. Future studies with larger, multi-site cohorts and validated patient satisfaction tools are needed to better understand the impact of telehealth etiquette training and to further guide its integration into health professions curricula.

## Introduction

Since the COVID-19 public health emergency, telehealth has expanded rapidly and remains a lasting component of healthcare delivery (1). Ndwabe et al. (2) reported on global utilization of telehealth by country and continent since the pandemic and summarized remarkable advancements in the adoption of telehealth (2). A report from the US Center for Medicare & Medicaid Services (CMS) in May of 2025 indicated that since the pandemic (i.e., 2022–2024), the proportion of patients insured through Medicare, typically adults aged 65 years and older, who chose a telehealth session when offered this modality, remained relatively stable, ranging from 25% to 29% over the observed period (3). Even as in-person sessions have resumed, many patients continue to choose telehealth, emphasizing the importance of high-quality virtual sessions. With the increased utilization of telehealth sessions, effective patient-provider communication must translate; however, many health professions education programs including allied health (4), nursing (5), and medicine (6) are not yet adequately preparing the future workforce in healthcare digital readiness. This is concerning because the future workforce is not being well trained in new telehealth communication skills that can impact patient satisfaction and healthcare outcomes. The term “telehealth etiquette,” (also known as webside manner) describes these skills. They are defined as the “unique behaviors beyond that of typical professional behaviors in order to conduct an effective telehealth visit or more importantly prevent a poor telehealth visit.” (7) *p* 255.

Given the current state of training, there is a need for health professions educators to expand clinical education beyond technical telehealth skills and reform their curricula to develop training focused on interpersonal communication. In fact, Shuy & Punjabi asserted in their 2022 call to action that educators must intentionally develop students' telehealth communication competency (8). Similarly, Serwe et al. in their 2020 telehealth student experiences scoping review concluded that future educational research should evaluate the impact of educational methods designed to teach students how to establish rapport with patients (9). Such training should enable students to understand the nuances of virtual vs. in-person sessions while developing empathy and nonverbal communication in the virtual environment (8, 10, 11). This is important because prior research of in-office healthcare sessions indicates that when the patient-clinician relationship is positive, patients understand their health better and are more likely to adhere to their treatment plans (12). Effective patient-provider communication improves patients' satisfaction and thereby positively influences their healthcare outcomes (13–15). Without targeted instruction and practice opportunities, the future healthcare workforce may lack the skills needed to provide empathetic, patient-centered care in telehealth.

Experiential learning in student-led university clinics allows students to practice and strengthen clinical skills, including interpersonal communication within safe and supportive supervised learning environments (16). A recent systematic review of student-led clinics reported the most common outcomes for students included improved interpersonal skills (including communication and patient-centered interactions), as well as professionalism (17). Similarly, Vargas et al. (18) reported on student improvement in patient-centered care and communication, and readiness for practice in their scoping review of the impact of interprofessional student-led clinics. As such, student-led clinics are valuable training environments for health professions programs to develop and pilot practical curricula in healthcare digital professional and communication readiness with patients.

In addition to real patient practice in student-led clinics, simulation-based education (SBE), often involving simulated participants (SP), people hired to portray patients, offers another type of experiential learning experience for students to develop their skills. SBE utilizes Kolb's theory to reinforce didactic learning in practice in risk-free, psychologically safe environments. While traditional engagement of SPs involves the SP and student working together in a simulated clinic room, SPs can also partake in telesimulations, simulations in which participants are separated geographically (19). Evidence supports the effectiveness of telesimulation. In two recent studies, SPs were engaged to portray the roles of patients in simulations focused on developing health professions students (20) and clinicians' (21) telehealth skills. In both studies, the engagement of SPs and the feedback they offered was effective in assessing the simulations' learning objectives. The study participants also benefited: the students indicated that they felt more confident, more knowledgeable, and more prepared to conduct telehealth sessions. The clinicians' results also indicated an improvement in confidence and skills for the associated learning objectives (21). Therefore, utilizing simulations with SPs to increase the non-technical skill training of health professions students in telehealth may be a viable solution.

Given the limited evidence-based communication and professionalism telehealth curricula despite the evidence that proves the importance of health professionals' communication and professionalism on health outcomes and patient satisfaction, we sought to understand the impact of an innovative multi-dimensional telehealth etiquette curriculum on students' telehealth etiquette skills and SP's “patient” satisfaction.

## Methods

### Theoretical framework

This pilot study seeks to compare baseline vs. intervention periods of the impact of telehealth etiquette training, integrating didactic instruction, experiential learning, and weekly self-reflections. Because of the scarcity of published literature on the impact of telehealth etiquette training with feedback from SPs, the researchers developed a novel, telehealth etiquette curriculum grounded in Kolb's experiential learning theory to develop telehealth skills in real clinical environments and telesimulations. This theory describes learning as a repeating cycle in which students grow through concrete experiences, reflecting on them, forming new understandings based on their reflections and debriefing, and then applying those insights in new situations. Kolb's experiential learning theory guided our curricular developments, such that we emphasized learning in the student-led clinic through a cycle of concrete experiences, reflection, conceptualization, and application (see the descriptions of the periods below) to support the students' integration of telesimulations and real patient care into a coherent, iterative skill-building process.

An IRB protocol was reviewed and approved by Mass General Brigham IRB (2024P003253). Students enrolled in clinical education practica in a health profession's pro bono clinical education center, where at least 25% of services were conducted via telehealth, were invited to participate. Recruitment occurred through an IRB-approved flyer posted on course websites two weeks before the semester, and an information session during which the principal investigator (PI) reviewed the study's aims, the details about each phase of the curriculum, the risks and benefits of participating, the informed consent process, and participant confidentiality. Conflicts of interest and potential coercion were proactively addressed in written and verbal communication, including explicit statements affirming that students' decisions to participate or not was voluntary and would not impact academic standing or grades now or in the future. In addition, the PI, who did not have a role in grading students shared that “data will not be reviewed until after the appeals period for grades has ended. During the data collection period, your data that is collected through the secure MGB-supported Research Electronic Data Capture (REDCap) software system and will be viewed only by the principal investigator and not by your faculty of record. After your grades have been submitted and the appeals period for grades has passed, only then, will the deidentified data be shared with the research team, in aggregate across student groups, in order for the team to analyze the results of the study”.

Eight of 15 eligible students (four speech-language pathology [SLP] students and four nurse practitioner [NP] students) chose to participate, and all eight students' voluntary consent was obtained prior to data collection. NP students participated in this 10-week curriculum in the 3rd of 4 semesters in their program while SLP students participated in the 3rd of 6 semesters for their program of study. On average, throughout the full semester, both cohorts were precepted with consistent preceptors. Pre-curriculum questionnaires completed by these students included a demographic survey (Table 1), a survey about their comfort with telehealth, Telehealth Comfort Scale (TCS) (22), and a survey about their telehealth etiquette knowledge, Telehealth Etiquette Knowledge Scale (TEKS) (23). A brief overview of the curriculum can be found in Table 2.

**Table 1:** Demographic data results.

ID	Master's degree program of study	Prior telehealth experience as the clinician	Prior telehealth experience as the patient	Number of weekly reflections the student completed during this study
1	Nurse practitioner	Yes	Yes	2 of 3
2	Nurse practitioner	Yes	No	3 of 3
3	Nurse practitioner	Yes	Yes	2 of 3
4	Nurse practitioner	Yes	Yes	0 of 3
9	Speech-language pathology	Yes	Yes	2 of 3
10	Speech-language pathology	No	Yes	3 of 3
11	Speech-language pathology	No	Yes	3 of 3
13	Speech-language pathology	No	Yes	3 of 3

**Table 2:** Overview of the multi-dimensional curriculum.

Phases of the curriculum	Curricular activities
Pre-Intervention (week 0)	Students completed pre-study surveys
Pre-Intervention (weeks 1–3)	Baseline period when students conducted routine precepted clinical practice in the student-led clinic. In post-session precepting meetings, preceptors did not emphasize telehealth etiquette instruction.
Pre-Intervention (week 4)	SP was extensively trained to understand her role, responsibilities, and portrayal expectations. She was also trained to use feedback tools.
	Students completed telehealth simulations with a SP portraying a caregiver of an Alzheimer's patient, focusing on rapport, interviewing, and professionalism. SP completed feedback tools.
Intervention (weeks 5 &6)	Students completed 4 short telehealth etiquette training modules covering curriculum relevance, best practices, and the TECC checklist.
Intervention (weeks 7–9)	Students applied TECC skills during precepted telehealth sessions and submitted weekly reflections on challenges, successes, and their impact on client-clinician relationships. In post-session precepting meetings, preceptors were encouraged to offer telehealth etiquette instruction, when applicable.
Post-Intervention (week 10)	Students completed post-simulations with the same SP with same feedback tools.
	Students completed post-study surveys.

### Pre-intervention period

The first three weeks of this 10-week study served as a baseline period, during which students conducted clinical practice sessions with pro bono clients receiving speech-language pathology and supportive counseling nursing services, both in-person and through videoconferencing-based telehealth. Faculty preceptors provided routine feedback typical of the student-preceptor relationship; no explicit emphasis was placed on telehealth etiquette. In week 4, students participated in telehealth simulations with the SP. In order to develop those cases and ensure the SP was adequately trained, the authors of this manuscript who have subject matter expertise in adult neurological communication disorders and mental health counseling developed the simulation. Using backwards design principles (24), they wrote the case (and the post-intervention case) grounded in curricular learning objectives. In accordance with Association of Simulated Participant Educators' Standards of Best Practice (25) domain three (SP training), the SP was trained for this specific case by receiving instructions about the simulation, the simulation script, and the feedback tools five days in advance. Leading up to the first simulation, the SP reviewed the contents, practiced the case on her own, asked questions about the case, and conducted a rehearsal with feedback from the PI the day prior to the simulation. The PI gave feedback on which aspects of the case to emphasize based on the student's discipline (SLP or nursing) as well as feedback on how to respond when students asked unanticipated questions. Additionally, the SP was trained to use two rater feedback tools, including a telehealth satisfaction survey developed for this study and hereto referred to as the Telehealth Satisfaction Survey (TSS). The TSS, created to assess proxy patient satisfaction of NP and SLP students' simulated sessions, drew on published surveys that best aligned with the telehealth satisfaction concepts we aimed to capture, in the absence of a single appropriate validated instrument. Items 1–5 were adapted from Hooshmand et al.'s survey on communication, picture quality, sound quality, privacy, and comfort with telehealth. Items 6–9 were adapted from Du and Gu (26, 27) who synthesized information from their systematic review into a 39-item satisfaction scale for telemedicine. From that survey, we adapted questions from the “overall satisfaction” section as those were most applicable to our curriculum. The SP was also taught to use the Telehealth Etiquette Competency Checklist (TECC) (28), which is a validated checklist of the telehealth etiquette skills that should be used during a telehealth session to promote a positive experience for patients. A complete list of the instruments used in this study can be found in Supplementary Materials. The day of the simulation, the SP and PI conducted a follow-up rehearsal to resolve any remaining ambiguities in the case and to ensure the SP was prepared to reliably use the rater feedback tools. Each simulation paired one student with the SP and ended with the SP providing feedback on telehealth etiquette objectives.

Kolb's learning cycle was initiated at this stage through students' concrete experience or feeling of engaging in the telehealth simulation. Students participated in 10–15-minute telehealth simulations using Zoom with the SP portraying the spouse of a patient who was recently diagnosed with Alzheimer's disease. The learning objectives were to establish rapport, demonstrate professionalism, and interview the caregiver to identify the presenting problem and therapy goals. Best practice standards in simulation pedagogy were employed during the simulation prebrief in which students reviewed a fiction contract (which invited students to engage fully in the simulated scenario by setting aside disbelief (29)) a confidentiality statement, the Basic Assumption, (which is a core value to support psychologically-safe learning environments (30)) an overview of case logistics, simulation goals, and feedback procedures. Reflective observation and abstract conceptualization of these new skills was also initiated.

### Intervention period

During weeks five and six, in addition to the clinical practice sessions students were conducting as part of their regular student-led clinic sessions, they also progressed in Kolb's cycle returning to the “concrete experience” when they participated in asynchronous online telehealth etiquette training consisting of four training modules, each lasting 5–10 min. The first module introduced the curriculum and explained why telehealth etiquette training is timely, relevant, and important to patient satisfaction. Students were also provided with a review of the evidence supporting patient satisfaction and health outcomes. The second module presented images and video examples of both effective and ineffective etiquette, including a model telesimulation that demonstrated best practices. The third module included a brief overview of a recent telehealth etiquette scoping review (31) and introduced the Telehealth Etiquette Competency Checklist (TECC (28)), which students were expected to apply during upcoming telehealth sessions. The final module reviewed the four TECC domains (i.e., technology, environment, confidentiality, and communication), as well as each of the 20 TECC skills (e.g., “ensure lighting in your environment adequately highlights your face” and “pause mindfully between speakers to minimize audio interruptions”). Students were also provided with a list of 17 additional optional telehealth etiquette resources for self-study.

In weeks seven through nine, students progressed in Kolb's cycle into the “abstract conceptualization, active experimentation” stages while conducting their clinical practice sessions in their respective disciplines (SLP or NP). During the videoconferencing-based telehealth sessions, they utilized the TECC checklist to support recall and application of skills. To encourage deliberate reflection and transfer of clinical skills to practice (in alignment with best practices for reflection that are clinically contextual, repeated, and incorporated into precepting conversations), students submitted weekly written responses with reference to their completed TECC checklists and their clinical notes from that week (32). The questions included: Which TECC skills were more difficult to consistently use this week and why? Which TECC skills were easy to recall and use this week and why? Do you think using the TECC skills this week has helped to promote a positive client-clinician relationship between you and your client? If yes, which skills do you feel contributed most positively to the client-clinician relationship? If not, why do you feel these skills have not promoted a positive client-clinician relationship?.

### Post-intervention

In week ten, students participated in post-intervention simulations with the same SP from the pre-intervention simulations. The procedures mirrored the earlier simulation: the SP and students received case details in advance, goals for the session which included progress evaluation with goal refinement, and the SP was retrained on the scenario and rating tools. In the fictional contract, this encounter was framed as a follow-up session occurring “three months after the initial visit.” Again, the students were rated using the same rater feedback tool, and the SP provided the students with verbal and written feedback following the simulations. Students completed the same surveys post-intervention while blinded to their pre-intervention responses. Student reflections and the simulation debriefs engaged students again in the concrete experience and reflective observations stages of Kolb's cycle.

### Data analysis

At the conclusion of the data collection period, the research team consulted with an expert in health professions quantitative methods to support the quantitative analysis. Following discussion, we agreed that the sample size was small which could result in low power to detect effects of interest. Still, the Wilcoxon signed rank test would be a feasible test for us to understand the data, since it is a non-parametric test with minimal assumptions. Additionally, computing the effect size adds additional information beyond the *p*-values alone. All computations were conducted within R Statistical Software (R Core Team, May 25, 2025; v4.3.1) using coin (33) and rstatix (34) packages. Effect size was calculated as Z statistic divided by square root of the sample size (N) (*Z*/*N*−√) (35). We repeated the test using a simpler sign test, which does not have the same symmetry assumption of the signed rank test, and found that the significance and interpretation of the *p*-values did not change, indicating that this assumption is unlikely to have an outstanding impact on the results.

The journal entries were also analyzed at that time by two independent raters on this research team (from the fields of SLP and nursing) to conduct a content analysis, according to Braun and Clarke's six-phase framework: (1) familiarization with the data, (2) generating initial codes, (3) searching for themes, (4) reviewing themes, (5) defining and naming themes, and (6) producing the final report (36). Multiple codes were permitted per reflection and since the students were asked to identify specific skills among the 20 from the TECC to answer the questions, minimal interpretation to search for themes was required to understand the meanings within their reflections. For instance, when asked which of the skills were easiest to recall and use, one student wrote “reducing distractions, simplifying background” which directly corresponds to TECC skills #3 (“reduce distractions [e.g., email notification noises] in your environment that could impact the quality of the visit”) and #6 (“simplify the background in your room to limit visual distractions [e.g., lots of wall art]”). Therefore, the raters defined and named the themes in alignment with associated TECC skills. Because of the way the questions were asked, all responses fit one or more of the TECC skills. We then counted the frequency of these responses. Using Excel to organize the data and trustworthiness methods such as transparency in the analysis process with clear audit trails and blinded two-person ratings, 100% interrater reliability was achieved through percent agreement calculations.

## Results

Descriptive statistics (Table 3), including means and standard deviations and Exact Wilcoxon-Pratt Signed-Rank Tests with associated effect sizes (Table 4) were used to compare the pre- and post-intervention data for all participants. Demographic characteristics (Table 1) indicated that all NP students reported prior experience conducting telehealth sessions as clinicians, while only one of the four SLP students had similar experience. Nearly all students (*n* = 7) had prior experience as telehealth patients. NP students conducted 2 telehealth sessions per week (approximately 87% of their total caseload) while SLP students conducted 5 telehealth sessions per week (approximately 60% of their total caseload). These differences reflect the various treatment plans (i.e., a client participating one vs. two times per week), as well as varying student schedules. Qualitative outcome data were derived from weekly student self-reflections regarding application of telehealth etiquette skills (see Table 5).

**Table 3:** Descriptive statistics results .

Time point	Survey	Range of scores	Mean scores (SD)
Pre	Telehealth Comfort Scale (22)	26–36	28.75 (3.49)
Post	Telehealth Comfort Scale (22)	26–42	32.50 (6.72)
Pre	Telehealth Etiquette Knowledge Scale (23)	41–57	52.88 (5.49)
Post	Telehealth Etiquette Knowledge Scale (23)	44–57	54.50 (4.38)
Pre	Telehealth Satisfaction Survey	30–37	34.13 (2.10)
Post	Telehealth Satisfaction Survey	24–35	32.13 (3.76)
Pre	Telehealth Etiquette Competency Checklist (28)	4–10	8.25 (1.83)
Post	Telehealth Etiquette Competency Checklist (28)	5–16	11.88 (3.44)

**Table 4:** Exact Wilcoxon-pratt signed-rank test inferential statistical results.

Outcome measure	Result of test	Effect size (small: *r* = 0.1–0.29; medium: *r* = 0.3–0.49; large: *r* = >0.5) (40)
Telehealth Comfort Scale	*Z* = 1.4055; *p*-value = 0.1875	*r* = 0.497 moderate
Telehealth Etiquette Knowledge Scale	*Z* = 1.4924; *p*-value = 0.2031	*r* = 0.528 large
Telehealth Satisfaction Survey	*Z* = −1.6223, *p*-value=0.1406	*r* = 0.574 large
**Telehealth Etiquette Competency Checklist** ^a^	***Z*** **=** **2.5236; *p*-value = 0.007812**	*r* = **0.892 large**

a indicates statistically significant finding.

**Table 5:** Qualitative content analysis with frequency counts.

Skills	TECC (28) domain with associated skill(frequency count for this topic across students for the three weeks this data was collected)	Representative quotes
Easiest skill to implement	Environment: Simplify the background in your room to limit visual distractions. (5/18)	ID#1: “Simplifying the background was the easiest just because I was working from school, where all the walls are plain white.”
	Communication: Verbally wrap up the visit by summarizing and providing next steps. (5/18)	ID#2: “Background, wrapping up the session, scheduling next appointment.”
Most impactful skill on positive relationship establishment	Communication: Use clear nonverbal communication to express empathy in the absence of physical touch. (8/18)	ID# 9: “Adherence to a routine involving checking environment, maintaining eye contact, maintaining empathy.”
	Environment: Reduce distractions in your environment that could impact the quality of the visit. (6/18)	ID#13: “Using active listening, removing background noise, trying to provide compassion through zoom.”
	Communication: Engage in eye contact through a virtual platform by directing your gaze at the camera rather than the screen. (6/18)	ID# 10: “Engage in eye contact through a virtual platform by directing their gaze at the camera rather than the screen.”
	Communication: Verbally wrap up the visit by summarizing and providing next steps. (6/18)	ID #2: “Wrapping up the session appropriately.”
Most difficult skill to implement	Communication: Engage in eye contact through a virtual platform by directing your gaze at the camera rather than the screen. (6/18)	ID#1: “Eye contact is difficult, and I'm actually not confident that it is even perceived the same way through video chat, especially with younger patients who have grown up using video chat.”
	Confidentiality: Request that the patient introduce anyone present in his/her location. (4/18)	ID#3: “Looking at the camera instead of the screen and directly requesting that they are in a private location and for them to introduce anyone else present at the location. These aspects of telehealth feel awkward.”
		ID#11: “Requesting that the patient introduce anyone present in their location. Requesting that the patient participate in the telehealth visit from a private location. Since I see them on Zoom 2x a week, it's not something I address when I see them.”

Students' increased use of telehealth etiquette skills on the TECC (28) was statistically significant with a large effect size (*z* = 2.52, *p* = .008, *r* = 0.892), with a mean score change from 8.25 to 11.88. These objective findings aligned with students' self-perceived improvements in telehealth etiquette knowledge and application.

Growth in students' telehealth comfort was also noted: while the amount of change was not statistically significant, the change had a moderate effect size (*r* = 0.497): students' mean TCS scores increased from 28.75 pre-intervention to 32.50 post-intervention (25). A full point increase was observed on the prompt, “I am able to gather information equally as effectively via telehealth as I am able to in person.” SLP students reported larger gains than NSG students (group mean change from 7 vs. 0.5). Minimal change was observed (pre-intervention mean = 4.75; post-intervention mean = 5.0) for telehealth etiquette knowledge (using TEKS (26)), likely reflecting a ceiling effect as most students already endorsed high agreement with knowledge items. For example, all students strongly agreed with the pre-intervention statement, “When transferring patient information, you must follow HIPAA guidelines.” While the change was minimal, the effect size was large (*r* = 0.528).

The SP's satisfaction with telehealth, using the 9-item TSS decreased slightly from pre-intervention (mean = 3.79) to post-intervention (mean = 3.57). While this change did not reach the level of statistical significance, the effect size was large (*r* = 0.574). This negative change was larger when evaluating NP students compared to SLP students (group mean change of −3.57 vs. 0.5) though the amount of change across students is not statistically significant and is considered an exploratory finding, given the small sample. However, post-intervention ratings showed improvement on specific items related to session comparability and telehealth recommendation, suggesting nuanced perceptions rather than a uniform decline in satisfaction. The SP specifically noted post-intervention, “the telehealth visit was as good as an in-person visit” and “I would recommend telehealth as a good option for other patients.”

### Qualitative content analysis

Through the three weeks of journaling, all students consistently replied that using the telehealth etiquette skills did promote a positive relationship with their clients (18/18 reflection journals). Table 5 shows the results of the qualitative content analysis from the student journal entries. The most frequently cited skills, reported as the easiest skills to recall and implement during sessions, identified through content analysis, were simplifying background visuals (*n* = 5) and clearly closing sessions (*n* = 5). Students also identified that the skills contributing most positively to client-clinician rapport were expressing nonverbal empathy (*n* = 8), reducing environmental distractions (*n* = 6), maintaining eye contact (*n* = 6), and providing session closure (*n* = 6). They stated that they had consistent challenges with maintaining eye contact via camera (*n* = 6).

## Discussion

The purpose of this pilot was to examine the impact of a telehealth etiquette curriculum on health professions students' development of communication and professional healthcare digital readiness skills within a precepted student-led clinic. This pilot study addresses a gap in the literature on telehealth communication training for health professions students, responding to Shuy and Punjabi's (8) call to intentionally develop telehealth communication competency (8) and to Serwe et al.'s recommendation to evaluate educational methods that support nonverbal empathic telehealth communication with patients (9). This study also aimed to develop students in enhancing patient satisfaction. We evaluated the impact of the curriculum on health professions students’ application of new skills, using Kolb's experiential learning cycle in a student-led clinic, which integrated precepted clinical practice, simulations, asynchronous trainings, self-reflections and feedback. A secondary aim was to examine the effects of this training on patient-provider satisfaction, given the established link between this relationship and patient outcomes (14, 15). NP and SLP students participated in pre- and post- intervention telesimulations with an SP who observed their telehealth etiquette skills, rated satisfaction, and provided feedback.

To address the first aim, quantitative findings suggest that the curriculum effectively improved students' application of telehealth etiquette skills. Student's self-perceived growth was supported by the SP's objective ratings on the TECC, which showed statistically significant improvement from pre- to post-intervention. Notably, students consistently improved in reducing environmental distractions, a skill they also identified in their journal reflections as easiest to adopt. This finding, triangulated by both quantitative and qualitative analyses, suggests that teaching students to manage their environment may be an early and accessible developmental target to foster presence and attentiveness, traits previously identified as critical for practice-ready patient-centered clinicians (10). Improvement in confidentiality-specific skills were also observed, highlighting their importance in establishing trust and rapport, noted as other traits important for the development of a positive patient-provider relationship and a critical need for our future workforce (10, 28). All students self-reported an improvement in their comfort using telehealth (on TCS), and while the change is promising with moderate effect size, the findings likely have limited statistical power because of the small sample size. The change could suggest that with increased practice in telehealth, students increase their comfort. Another observation was the degree of change between NP and SLP students. This variation may reflect differences in practice opportunities (NP students averaged two telehealth sessions per week, compared to five for SLP students), differences in prior experience with telehealth (NPs had all provided telehealth before which may have impacted their learning capacity in this curriculum), and differences in preceptor feedback. Drawing on the research in practice dosage and retrieval, and the importance of preceptor feedback on students' growth, the variability in these learning factors could explain the exploratory group-level differences in telehealth comfort levels (37, 38).

Students' weekly reflections highlighted the communication skills (expressing empathy nonverbally, maintaining eye contact, and providing clear session closure) that they felt had the most positive impact on client relationships and interactions. As stated earlier, they also highlighted the skill of minimizing distractions. These reflective insights suggest that participating in this telehealth etiquette curriculum supported the students’ development of the traits they will need as they enter the workforce to promote patient-centered healthcare professionals and satisfied patients (8, 11, 39). They also align with previous findings of communication and professionalism improvements within student-run clinical experiences (17, 18). Lastly, findings from this study also extend prior research into the benefits of participating in student-run telehealth clinical experiences from improvements in technology and management skills to the distinct improvement of telehealth etiquette skill application (18).

While this study shared similar positive outcomes of SP engagement for simulation participants' telehealth competency development, as was seen by Nash et al. (21) and Taylour & Coulter (20), this study's second aim sought to expand the evidence base for SP engagement as a proxy for patients' satisfaction ratings. Despite students' perceived improvements in clinician-client relationships from pre- to post-intervention, the SP's satisfaction ratings did not show a statistically significant change over the course of the study. Several factors may explain this discrepancy, including the use of a non-validated tool for measuring patient satisfaction and the fact that the SP was not blinded to the research questions, which raises the possibility of unconscious bias. Other potential interpretations include insufficient sensitivity of the non-validated TSS tool. While the SP ratings indicated lower satisfaction with telehealth overall post-intervention, ratings for the post-intervention NP group were notably lower than those for the post-intervention SLP group, a difference that may reflect the varying practice opportunities and preceptor feedback that were specific to the two discipline cohorts during the study.

This pilot study has several limitations. The small sample size and single-site design limit generalizability, and only one simulated participant (SP) served as the sole rater. The SP was not blinded to the study aims, which may have introduced risk of expectancy effects and rating bias, and the telehealth satisfaction tool used was adapted from prior work but had not undergone formal validation. The absence of psychometric evidence for the satisfaction tool constrains interpretation of non-significant findings and the SP's risk potential is a central limitation as all of the primary outcomes depended on her evaluations. Differences in student exposure (i.e., NP students had fewer telehealth sessions than SLP students, one NP student had no telehealth experience prior to participating in this curriculum) and variability in engagement with the asynchronous modules may have influenced outcomes. Additionally, nursing students were consistently scheduled earlier in the week and speech-language pathology students later, creating the possibility of a primacy or recency effect on SP ratings.

Finally, while this project included students from two professions (NP and SLP), it was not a fully interprofessional experience as students worked primarily 1:1 with their clients and 1:1 with their preceptors. In future iterations, interprofessional training and shared debriefing would likely strengthen outcomes for all participants because students would benefit from learning together in their integration of telehealth etiquette skills. In addition, the differences observed between NP and SLP students highlight that robust and repeated training is needed to reinforce skill development and ensure consistency across disciplines, though these conclusions remain exploratory.

Future studies should address these limitations by recruiting larger, multi-site cohorts, incorporating multiple blinded SP raters, validating satisfaction measures, and ensuring consistent participation across student groups. Such studies would further clarify the impact of telehealth etiquette training and help guide integration of these competencies into scalable health professions curricula.

## Conclusions

This pilot study revealed that health professions students improved their use of telehealth etiquette skills following a structured Kolb's-aligned curriculum that combines online modules, telesimulation, and reflection. Students applied skills such as eye contact, empathy, and clear closure more consistently and described these as making a meaningful difference in their relationships with clients.

While overall satisfaction ratings from the simulated participant did not change significantly, the findings highlight both the promise and the complexity of measuring outcomes related to patient experience. Differences between NP and SLP students suggest that practice opportunities and feedback may shape outcomes as much as the training itself.

As a small, single-site study with one SP rater, these results should be interpreted as preliminary. Still, they point toward the value of teaching telehealth etiquette in health professions education. Future work should expand to larger, interprofessional groups, incorporate blind raters and validated tools, and explore how etiquette training influences real patient outcomes.

## Data Availability

The raw data supporting the conclusions of this article will be made available by the authors, without undue reservation.
