# Supplementary material for: Professionalism on camera: results of a telehealth etiquette pilot curriculum on health profession students’ competency
Source: Front Health Serv. 2026 May 25;6:1840148. doi: 10.3389/frhs.2026.1840148 (PMC13243253; doi:10.3389/frhs.2026.1840148)
Supplement: Supplementary file 1 [file Supplementaryfile1.pdf]

Supplementary Material

Instruments used during the study

1. **Telehealth Comfort Scale** (Dadlani et al., 2023)[22]
   - psychometric properties of the scale not stated by Dadlani et al.
   - rating scale of 1-5: 1=strongly disagree; 5=strongly agree

2. **Telehealth Etiquette Competency Checklist (TECC)** (Pittmann et al., 2024)[28]
   - scale's Content Validity Index = 0.98.
   - response options: observed (1) or not observed (0), adapted for completion by simulated participant

3. **Telehealth Etiquette Knowledge Scale** (Rutledge et al., 2020)[5]
   - internal consistency of scale a=0.862 (n=407).
   - rating scale of 1-5: 1=strongly disagree; 5=strongly agree

4. **Telehealth Satisfaction Scale**
   - tool is not validated
   - tool is adapted from Hooshmand et al., 2021 [items 1-5][26] and Du & Gu, 2024 [items 6-9])[27]
   - rating scale of 1-5: 1=strongly disagree; 5=strongly agree
