# Supplementary material for: Professionalism on camera: results of a telehealth etiquette pilot curriculum on health profession students’ competency
Source: Front Health Serv. 2026 May 25;6:1840148. doi: 10.3389/frhs.2026.1840148 (PMC13243253; doi:10.3389/frhs.2026.1840148)
Supplement: Supplementary file 2 [file Table1.docx]

Supplementary Material

**Instruments used during the study**

1. **Telehealth Comfort Scale** (Dadlani et al., 2023)^22^

- psychometric properties of the scale not stated by Dadlani et al.
- rating scale of 1-5: 1=strongly disagree; 5=strongly agree

1. **Telehealth Etiquette Competency Checklist (TECC)** (Pittmann et al., 2024)^28^

- scale’s Content Validity Index = 0.98.
- response options: observed (1) or not observed (0), adapted for completion by simulated participant

1. **Telehealth Etiquette Knowledge Scale** (Rutledge et al., 2020)^5^

- internal consistency of scale a=0.862 (n=407).
- rating scale of 1-5: 1=strongly disagree; 5=strongly agree

1. **Telehealth Satisfaction Scale**

- tool is not validated
- tool is adapted from Hooshmand et al., 2021 [items 1-5]^26^ and Du & Gu, 2024 [items 6-9])^27^
- rating scale of 1-5: 1=strongly disagree; 5=strongly agree
